# Evaluation of Antioxidant, Antimicrobial and Tyrosinase Inhibitory Activities of Extracts from *Tricholosporum goniospermum,* an Edible Wild Mushroom

**DOI:** 10.3390/antibiotics9080513

**Published:** 2020-08-13

**Authors:** Paola Angelini, Roberto Venanzoni, Giancarlo Angeles Flores, Bruno Tirillini, Giustino Orlando, Lucia Recinella, Annalisa Chiavaroli, Luigi Brunetti, Sheila Leone, Simonetta Cristina Di Simone, Maria Chiara Ciferri, Gokhan Zengin, Gunes Ak, Luigi Menghini, Claudio Ferrante

**Affiliations:** 1 Department of Chemistry, Biology and Biotechnology, University of Perugia, 06122 Perugia, Italy; roberto.venanzoni@unipg.it (R.V.); giancarlo.angelesflores@unipg.it (G.A.F.); 2 Department of Biomolecular Sciences, University of Urbino, 61029 Urbino, Italy; bruno.tirillini@uniurb.it; 3 Department of Pharmacy, University “G. d’Annunzio” of Chieti-Pescara, 66100 Chieti, Italy; giustino.orlando@unich.it (G.O.); lucia.recinella@unich.it (L.R.); annalisa.chiavaroli@unich.it (A.C.); luigi.brunetti@unich.it (L.B.); sheila.leone@unich.it (S.L.); disimonesimonetta@gmail.com (S.C.D.S.); mariachiara.ciferri@outlook.it (M.C.C.); luigi.menghini@unich.it (L.M.); claudio.ferrante@unich.it (C.F.); 4 Department of Biology, Science Faculty, Selcuk Universtiy, Campus, Konya, Konya 42130, Turkey; akguneselcuk@gmail.com

**Keywords:** *Tricholosporum goniospermum*, antimicrobial activity, scavenger-reducing activity, anti-tyrosinase activity

## Abstract

*Tricholosporum goniospermum* (Bres.) Guzmán ex T.J. Baroni is an excellent edible mushroom whose compounds and biological properties are still unknown. In this study, n-hexane, ethyl acetate and methanol extracts from fruiting bodies and liquid-cultured mycelia were compared for the analysis of phenolic compounds, the evaluation of scavenger (DPPH, ABTS) and reducing (CUPRAC, FRAP) activities, and the enzyme inhibition of α-amylase, acetylcholinesterase (AChE), butyrylcholinesterase (BChE) and tyrosinase. Additionally, *T. goniospermum* extracts were evaluated for antibacterial and antimycotic activities against Gram+ and Gram− bacteria, and clinical yeast and fungal dermatophytes. Finally, based on the extract content in phenolic compounds, in silico studies, including the docking approach, were conducted to predict the putative targets (namely tyrosinase, lanosterol-14-α-demethylase, the multidrug efflux system transporters of *E. coli* (mdtK) and *P. aeruginosa* (pmpM), and *S. aureus* β-lactamase (ORF259)) underlying the observed bio-pharmacological and microbiological effects. The methanolic extract from mycelia was the richest in gallic acid, whereas the ethyl acetate extract from fruiting bodies was the sole extract to show levels of catechin. Specifically, docking runs demonstrated an affinity of catechin towards all docked proteins, in the micromolar range. These in silico data are consistent, at least in part, with the highest activity of ethyl acetate extract as an antimicrobial and anti-tyrosinase (554.30 mg KAE/g for fruiting bodies and 412.81 mg KAE/g for mycelia) agent. The ethyl acetate extracts were also noted as being the most active (2.97 mmol ACAE/g for fruiting bodies and 2.25 mmol ACAE/g for mycelia) on α-amylase. BChE inhibitory activities varied from 2.61 to 26.78 mg GALAE/g, while the tested extracts were not active on AChE. In conclusion, all mushroom extracts tested in this study had potent antimicrobial activities. Particularly, among the tested extracts, the ethyl acetate extract showed the highest efficacy as both an antimicrobial and anti-tyrosinase agent. This could be related, albeit partially, to its content of catechin. In this regard, the bioinformatics analyses showed interactions of catechin with tyrosinase and specific microbial proteins involved in the resistance to chemotherapeutic drugs, thus suggesting innovative pharmacological applications of *T. goniospermum* extracts.

## 1. Introduction

Fungi represent some of the principal components of biodiversity in all terrestrial habitats [1,2]. They are important decomposers and recyclers of organic materials; they positively or negatively interact with plant roots in the rhizosphere or with above-ground plant components [3,4,5,6,7].

For centuries, mushrooms, the fruiting bodies of macroscopic filamentous fungi that grow above the ground, have been a part of the human diet and used as both food and medicine [8]. They contain minerals, vitamins and nutritive compounds such as proteins and polysaccharides and have a low fat content [9]. Mushrooms are also appreciated as a delicacy. They have many flavors and nutrient characteristics that make them an ideal addition to many dishes. Their texture and umami or savory flavor properties make them a suitable substitute for meat [8].

While the usage of medicinal mushrooms has a long tradition in Eastern countries, in the Western world it has increased only slightly over the last few decades [10,11,12].

Mushrooms have been found to have potent biological activities such as anti-bacteria, anti-fungal, antitumor, anti-inflammatory, anti-hepatotoxic, cardio-tonic, cholesterol level lowering, antiviral and immune-modulatory activities [13,14,15]. The enormous structural diversity of natural compounds originated from mushrooms offers opportunities for discovering new drugs [16].

Antimicrobial resistance (AMR) presents one of the biggest challenges to global public health, and it was estimated to have claimed 700,000 lives globally in 2014. Furthermore, it has been predicted that its attributable mortality will hit 10 million by 2050 if measures are not taken to tackle it. Thus, it has become critical to safeguard the integrity of the antimicrobials currently in use, considering that the discovery of novel antimicrobials has stalled over recent decades [17]. Factors such as inappropriate use of antibiotics, inadequate infection prevention and control programs, limited laboratory capacity, poor surveillance, population growth and migration, as well as inadequate sanitation have compounded the problem of AMR [18]. Over the past decades, fungal infections have become a major problem in clinical practice, with immunocompromised patients being easily susceptible. Notably, systemic fungal infections are usually associated with high mortalities [19].

Infections by multidrug resistant isolates of *Trichophyton rubrum*, *Candida* spp., *Staphylococcus epidermidis*, *S. aureus*, *Streptococcus* spp., *Enterococcus* sp. and *Escherichia coli*, among others, became more and more frequent, stimulating the search for new antibiotics with novel mechanisms of action [20,21,22,23,24].

Considering the current problem of resistance to microbial drugs and the growing concern about opportunistic infections, there is an urgent need for alternative antimicrobial drugs that could be found in plants and mushrooms.

Over the past two decades, the health-promoting effect of mushrooms has attracted a lot of attention due to the wide range of secondary metabolites present in fruiting bodies and submerged culture [25,26]. In particular, about 270 species are now considered as potential therapeutic or preventative agents used to ensure human health [27].

To the best of our knowledge, the antioxidant and antimicrobial activities obtained from wild edible fungal extracts against pathogenic fungi and bacteria have been rarely explored. *Tricholosporum goniospermum* (Bres.) Guzmán ex T.J. Baroni (*Tricholomataceae*, Agaricales, Basidiomycota) is a wild mushroom mainly known from northern Europe and north-central Italy. It is considered a saprotroph that colonizes small soil patches near deciduous trees. Although it is considered an excellent edible mushroom, the biological properties of its fruiting bodies and mycelia extracts have not yet been studied [28]. It is apparent that, depending on the extraction solvent, the extracts from the same mushroom will differ in the composition of bioactive compounds.

Therefore, on the basis of these considerations, our present study aims to shed more light on this poorly understood mushroom. Our work is as follows: (1) clearly identify the fungus by means of macro- and micromorphological features as well as by molecular analysis (rDNA ITS sequence), (2) assess antioxidant properties in terms of radical scavenging, reducing, total antioxidant capacity, metal chelating and antimicrobial activities against major dermatophytes, yeasts and bacterial pathogens, (3) evaluate the enzymatic inhibitory effects related to chronic diseases, namely diabetes mellitus type II, neurodegenerative complications and skin disorders, and (4) make a comparison between different extraction solvents used to prepare the samples. A composition analysis was carried out as well, with the aim to investigate the mechanisms of action underlying the observed biological effects. In this regard, an in silico study was also conducted towards selected human and microbial target proteins, namely tyrosinase and multidrug efflux system transporters of *E. coli* (mdtK), *P. aeruginosa* (pmpM) and *S. aureus* β-lactamase (ORF259).

## 2. Results

### 2.1. Molecular Identification of the T. goniospermum Strain

**Figure 1:**
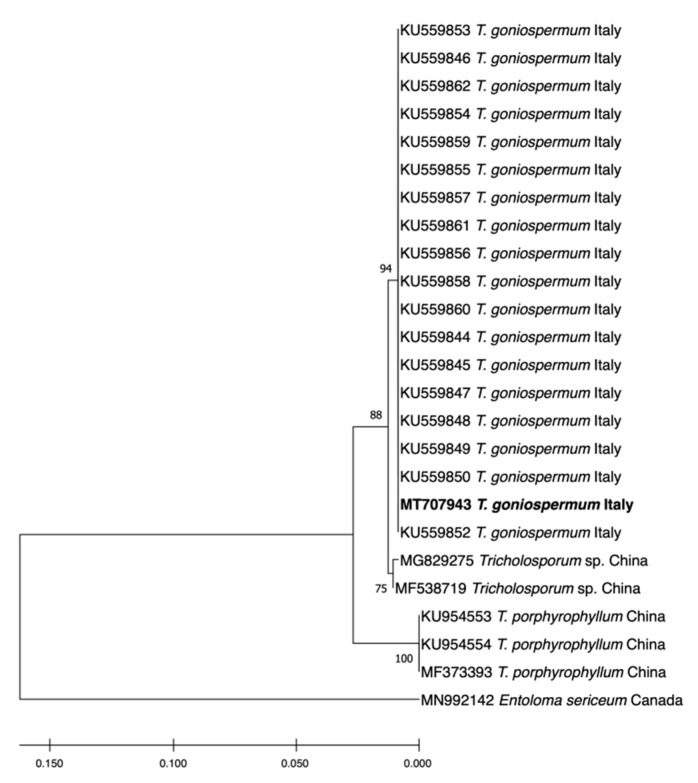
Maximum-likelihood (Tamura-Nei model) tree showing the phylogenetic position of the *Tricholosporum goniospermum* PeruMyc2084 isolate used in this study (corresponding to MT707943). We used sequences representing different *Tricholosporum* species and *T. goniospermum* samples. Both GenBank accession numbers and geographical origins are reported. Bootstrap values (expressed as percentages of 1000 replications) are shown at branch points.

The internal transcribed spacer (ITS) region of sample PeruMyc2084 was amplified through PCR and sequenced. A search with the BLAST confirmed that the samples belonged to *T. goniospermum*, as it showed a close match (100% sequence identity) with isolates of this species. In more detail, the phylogenetic analysis showed that the PeruMyc2084 strain belongs to the *T. goniospermum* clade (Figure 1).

### 2.2. High Performance Liquid Chromatography (HPLC) Analysis

**Figure 2:**
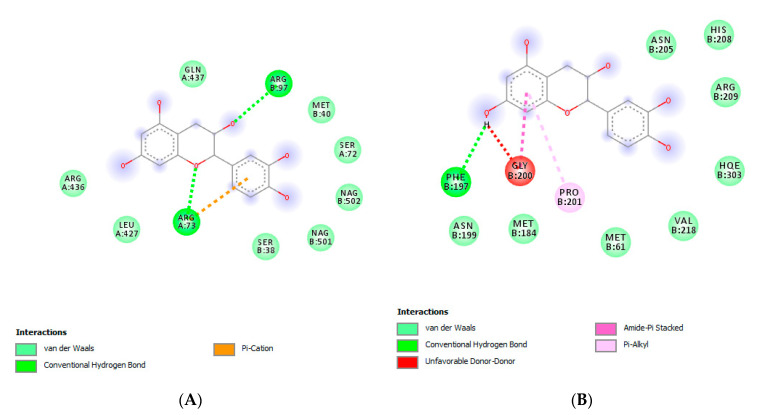
Putative interaction between catechin and human tyrosinase-related protein 1 (PDB: 5M8P). The free energy of binding (ΔG) and affinity (Ki) are −6.7 kcal/mol and 12.4 µM, respectively (**A**). Putative interaction between catechin and tyrosinase from *Bacillus megaterium* (PDB: 5I3B). The free energy of binding (ΔG) and affinity (Ki) are −6.6 kcal/mol and 14.7 µM, respectively (**B**).

The HPLC-fluorimetric analysis focused on selected phenolic compounds, namely gallic acid, catechin, epicatechin and resveratrol. The results indicated that the *T. goniospermun* methanolic extract from mycelia was the richest in gallic acid, whereas the *T. goniospermum* ethyl acetate from the fruiting body displayed the best qualitative profile, alongside low levels of catechin (Table 1). Conversely, the *T. goniospermum* n-hexane extract from mycelia displayed the poorest quantitative profiles with regards to the selected phenolic compounds. The results of the HPLC-fluorimetric analysis were consistent with the colorimetric evaluations of total phenols (expressed as mg of gallic acid per g of dry extract) previously described, although a punctual description of the *T. goniospermum* phenolic profile is still lacking. According to colorimetric analyses, the ethyl acetate extract seems to be the most effective in terms of scavenger/reducing (Table 2) and enzyme inhibitory (Table 3) activities. Specifically, the anti-tyrosinase effect (Table 3) of this extract was significantly higher when compared to the other extracts, thus suggesting applications in skin disorders characterized by an increased tyrosinase activity. Therefore, a docking analysis was conducted on catechin, in order to investigate the mechanism of action of the extracts. The docking runs showed a good affinity of catechin towards the selected target proteins (Figure 2).

### 2.3. Antibacterial and Antifungal Activity

Table 4, Table 5 and Table 6 show the MIC ranges and geometric means of extracts and synthetic drugs (ciprofloxacin, fluconazole and griseofulvin) against the tested bacterial, yeasts and dermatophytes strains, respectively. All mushroom extracts showed antimicrobial activity within the concentration range tested, but with a wide variability in terms of potency and selectivity (Table 4, Table 5 and Table 6). Regarding bacteria, the strongest inhibition was observed for EA and MeOH mycelia extracts (MIC 0.099 mg mL^−1^ against *E. coli*), while the methanol extract obtained by fruiting bodies resulted in actively reducing *B. cereus* growth (Table 1). Conversely, the n-hex fruiting bodies extract seemed to be the least effective against all the tested microorganisms (Table 4, Table 5 and Table 6). All bacterial strains were sensible to EA extracts from mycelia with MIC values lower than 0.315. The same extracts obtained from fruiting bodies gave similar results, but with slightly higher MIC values against *P. aeruginosa* (0.396 mg mL^−1^).

All tested extract results were actively inhibitory of fungal growth, but a huge variability was recorded between the most active (mycelia-EA on C. albicans YEPGA 6379: MIC 0.051) and the lesser ones (fruiting bodies-n-hex on *A. curreyi, A. insingulare, A. quadrifidum*: MIC > 1.0; fruiting bodies-MeOH on *A. insingulare*: > 1.0). Regarding dermatophytes, *A. crocatum* (CCF 5300) and *A. gypseum* (CCF 6261) were the most sensitive fungal species to mushroom extracts, with MIC ranges of 0.031–0.46 mg mL^−1^. The ethyl acetate extracts of mycelia were particularly active, showing the highest antifungal activity among all the tested strains.

All of the ten tested fungal strains showed an increased susceptibility toward the mushroom mycelia extract. Griseofulvin showed good activity in vitro against *A. gypseum* (CCF 6261) (MIC: 1–2 µg ml^−1^). It was not possible to determine whether the isolates were resistant to the griseofulvin as no breakpoints have yet been established.

**Figure 3:**
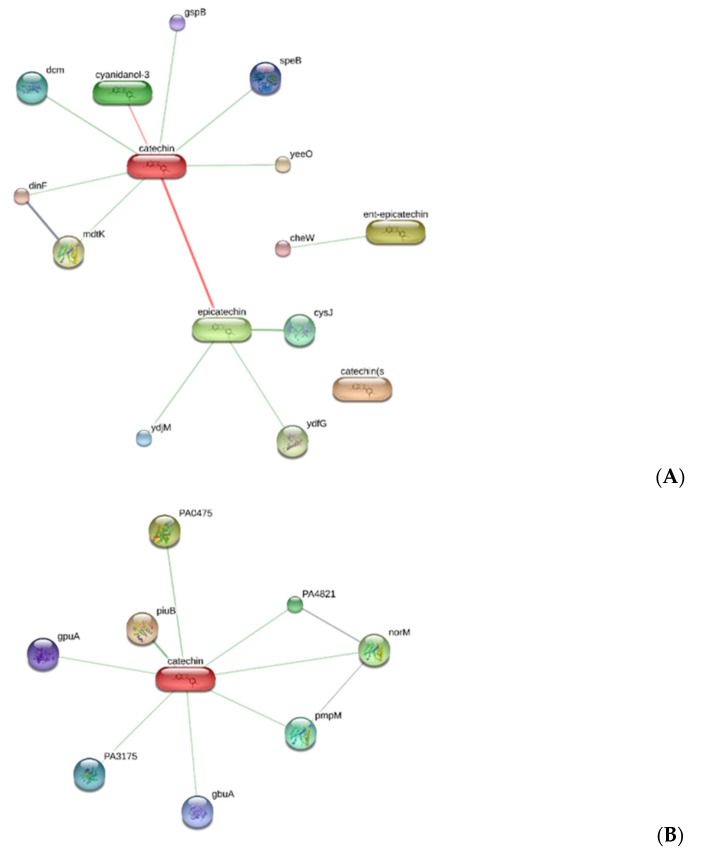

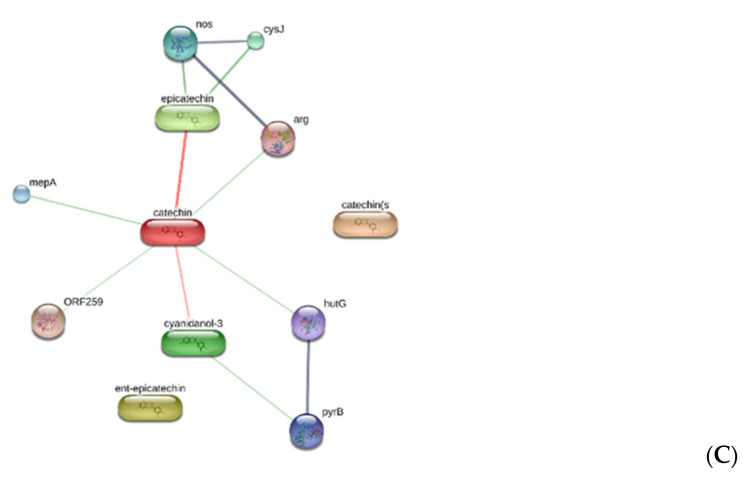
Component-target analysis underlying putative microbial proteins targeted by catechin, including the multidrug efflux system transporters of (**A**) *E. coli* (mdtK) and (**B**) *P. aeruginosa* (pmpM), and (**C**) *S. aureus* β-lactamase (ORF259).

Considering the promising antimicrobial activity by the ethyl acetate extract, a bioinformatics analysis was conducted through the platform STITCH, with the aim to identify microbial proteins that could be key targets of catechin. Specifically, the bioinformatics analysis predicted microbial proteins involved in the resistance to chemotherapic drugs, including the multidrug efflux system transporters of *E. coli* (mdtK) and *P. aeruginosa* (pmpM), and *S. aureus* β-lactamase (ORF259) (Figure 3).

**Figure 4:**
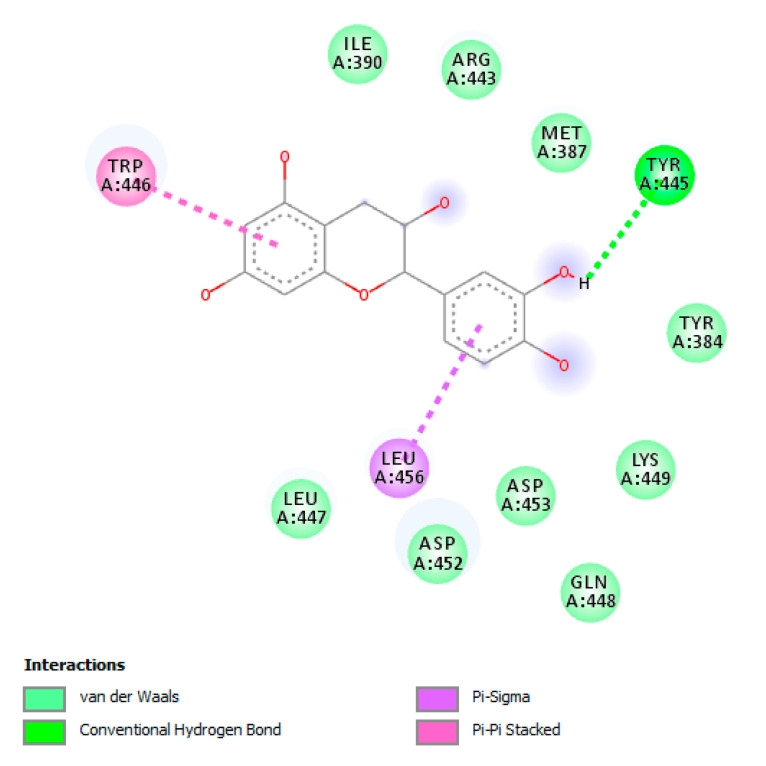
Putative interaction between catechin and the multidrug efflux system transporters of *E. coli* (mdtK) (PDB:3MKT). Free energy of binding (ΔG) and affinity (Ki) are −7.9 kcal/mol and 1.7 µM, respectively.

**Figure 5:**
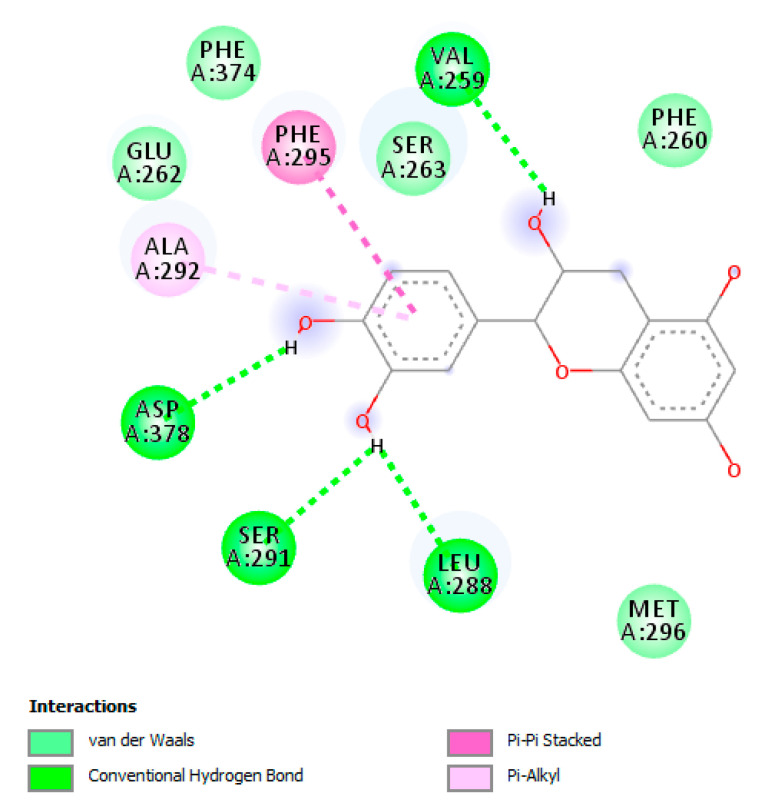
Putative interaction between catechin and the MATE transporter AtDTX14 (PDB:5Y50) as a homology model for describing the structure of the multidrug efflux system transporters of *P. aeruginosa* (pmpM). Free energy of binding (ΔG) and affinity (Ki) are −7.1 kcal/mol and 6.3 µM, respectively.

**Figure 6:**
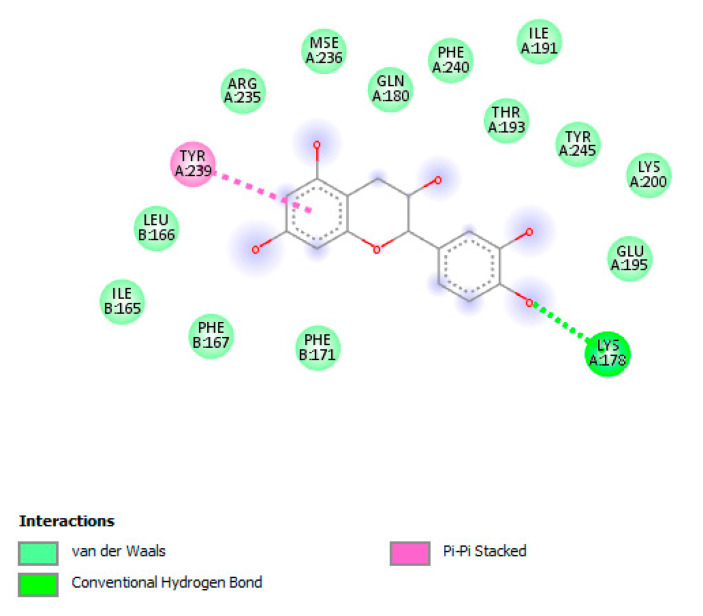
Putative interaction between catechin and *S. aureus* β-lactamase (ORF259) (PDB:3ESH). Free energy of binding (ΔG) and affinity (Ki) are −7.8 kcal/mol and 1.9 µM, respectively.

Meanwhile, the docking analysis showed the micromolar binding affinities of catechin towards these microbial proteins, further supporting the observed antibacterial activities exerted by the ethyl acetate extract (Figure 4, Figure 5 and Figure 6).

Furthermore, considering the involvement of bacterial and fungal opportunistic infections occurring in inflammatory skin disorders, including hyperpigmentation [29], the present findings support further biological investigations with the aim to confirm the application of the ethyl acetate extract of *T. goniospermum* in inflammatory dermatological disorders.

## 3. Materials and Methods

### 3.1. Mushroom Material

The fruiting bodies of the strain *T. goniospermum* PeruMyc2084 were collected in Pian Grande (Castelluccio di Norcia, PG, Italy) in June 2018. The Vaucher specimen (PeruMyc 2084) was identified on the basis of macromorphological and micromorphological features [28] and was deposited in the herbarium at the University of Perugia (Department of Chemistry, Biology and Biotechnology). In order to isolate mycelium in a pure culture, small pieces of context (about 10 mm^3^) were aseptically drawn from the fresh fruiting bodies and inoculated into Rose Bengal Chloramphenicol agar [30].

Incubation was performed in the dark at 25 °C for 14 days. Mycelium discs (5 mm in diameter) were then inoculated in each Petri dish [placed in the center of the Malt Extract Agar (Sigma-Aldrich, Milan, Italy) medium] under aseptic conditions. Free mycelium was obtained by culturing the isolated strain in 250 mL Erlenmeyer flasks containing 50 mL of 2% Malt Extract Broth (Sigma-Aldrich, Milan, Italy) supplemented with 1% glucose, pH 5.5. Four agar plugs (9 mm in diameter) were drawn from seven-day old cultures of *T. goniospermum* on Malt Extract Agar and inoculated into each flask. Incubation was performed statically at 25 °C for 10 days.

### 3.2. Molecular Identification

Genomic DNA was isolated from 15-day old mycelium grown in Malt Extract Agar (Sigma−Aldrich, Milan, Italy), according to [31]. The genomic DNA quality and quantity was evaluated by BIO-RAD model 200/2.0 Power Supply gel electrophoresis [0.8% agarose gel in 1 × TBE buffer (89 mM Tris, 89 mM boric acid, 2 mM EDTA, pH 7.6)] in the presence of SafeView Nucleic Acid Stain (NBS Biologicals) and a MassRuler DNA Ladder, Mix (Thermo Scientific, Lithuania), and visualized with Safe ImagerTM 2.0 Blue-Light Transilluminator Invitrogen (Thermo Fisher Scientific, Israel); DNA samples were consequently diluted by up to 10 μg/μL with nuclease-free water before PCR amplification. The internal transcribed spacer (ITS) region was amplified through polymerase chain reactions (PCRs) with ITS1F and ITS4 primers, according to Angelini et al. [31]. SimpliAmp Thermal Cycler AppliedBiosystems (Thermo Fisher Scientific, Singapore) was programmed as follows: one cycle of denaturation at 95 °C for 2.5 min; 35 cycles of denaturation at 95 °C for 20 s, annealing at 55 °C for 20 s and extension at 72 °C for 45 s; one final extension cycle at 72 °C for 7 min. Electrophoresis of PCR amplicons was carried out on 1.2% agarose gel as described above. The PCR-amplified ITS fragment was purified using the ExoSap-IT PCR Cleanup reagent (Affymetrix UK Ltd., High Wycombe, United Kingdom) and then sequenced by Macrogen Europe (Holland). The ITS sequence (GenBank accession no. MT707943) was checked for similarities by using the nucleotide Basic Local Alignment Search Tool (BLAST), available from the U.S. National Center for Biotechnology Information. The sequences showing the highest similarity with our query sequence were retrieved and aligned using Clustal W [24]. The phylogenetic analysis was performed by the Maximum Likelihood method and Tamura-Nei model, where gaps and missing data were eliminated (complete deletion option) [32]. Bootstrapping of the datasets was performed with 1000 replications. The *Entoloma sericeum* ITS sequence, retrieved from GenBank, was used as the outgroup. The phylogenetic analysis was conducted in Mega X [33,34].

### 3.3. Preparation of Mushroom Extracts

The fruiting bodies were finely ground and, after determination of humidity, were macerated in n-hexane for seven days at 20 °C (1:10 *w*:*v*). After filtration, the residue was macerated in ethyl acetate and then in methanol under the same conditions [seven days at 20 °C (1:10 *w*:*v*)]. The dry weight of the three extracts was determined. For biological tests, a known aliquot of each extract was brought to dryness at rotavapor (temperature 50 °C) and taken back into a known volume of DMSO. After washing with distilled water, the free mycelia were dried at 40 °C in a ventilated oven. After determination of residual moisture, they were extracted using the same extraction protocol used for the fruiting bodies.

### 3.4. Antioxidant and Enzyme Inhibitory Properties

The chemicals were purchased from Sigma−Aldrich (Darmstadt, Germany). They were: 2,2’-azino-, 1,1-diphenyl-2-picrylhydrazyl (DPPH), gallic acid, electric eel acetylcholinesterase (AChE) (type-VI-S, EC 3.1.1.7), horse serum butyrylcholinesterase (BChE) (EC 3.1.1.8), galantamine, acetylthiocholine iodide (ATChI), butyrylthiocholine chloride (BTChI) 5,5-dithio-bis(2-nitrobenzoic) acid (DTNB), tyrosinase (EC1.14.18.1, mushroom), amylase (EC. 3.2.1.1, from porcine pancreas), sodium carbonate, Folin−Ciocalteu reagent, hydrochloric acid, trolox, neocuproine, cupric chloride, ammonium acetate, kojic acid and acarbose. All chemicals were of analytical grade. The spectrophotometric measurements were performed by using a one microplate reader (Thermo Scientific Multiskan GO, Thermo Fisher Scientific Inc, Waltham, MA USA).

The total phenolic content was determined by using the colorimetric method (Folin−Ciocalteu method), and the result was expressed as the gallic acid equivalent. For the evaluation of the in vitro antioxidant activity, different assays were employed, namely DPPH, CUPRAC and FRAP. The detailed procedures regarding each method are described in previous works [35]. Briefly, in the DPPH assay, the methanolic DPPH solution (0.004%) was mixed with mushroom extract solution, after which the absorbances were recorded at 517 nm after a 30 min incubation. The CUPRAC assay measures the reduction of Cu^2+^ to Cu, and the absorbance of the final solution was read at 450 nm. Similar to the CUPRAC assay, the FRAP assay includes the transformation of Fe^3+^ to Fe^2+^, and the final absorbance was measured at 595 mm. The results were finally reported as mg Trolox equivalents (TE)/g extract.

To detect inhibitory effects on enzymes, we used colorimetric enzyme inhibition assays, and these assays included tyrosinase, α-amylase and cholinesterases. Some standard inhibitors (galatamine, kojic acid and acarbose) were used as positive controls, and the results were expressed as equivalents of these standards (GALAE, KAE and ACAE, respectively). Extracts were analyzed in triplicate [36].

### 3.5. HPLC Analysis

*T. goniospermum* n-hexane, ethyl acetate and methanolic extracts from fruiting bodies (2.3, 4.5 and 3 µg mL^−1^, respectively), and mycelia extracts (3.3 and 1.5 μg mL^−1^) were analyzed for a phenol quantitative determination using a reversed-phase HPLC-fluorimeter in gradient elution mode. The analyses were carried out by using a liquid chromatograph (MOD. 1525, Waters Corporation, Milford, MA, USA) equipped with a fluorimetric detector (MOD. 2475, Waters Corporation), a C18 reversed-phase column (AcclaimTM 120, 3 μm, 2.1 × 100 mm, Dionex Corporation, Sunnyvale, CA, USA) and an on-line degasser (Biotech 4-CH degasi compact, LabService, Anzola Emilia, Italy). The gradient elution was achieved by a mobile phase consisting of methanol/acetic acid/water (10:2:88, *v*/*v*) as solvent A and methanol/acetic acid/water (10:2:88, *v*/*v*) as solvent B, in agreement with already published papers [37,38]. Accordingly, λ_ex_ = 278 nm and λ_em_ = 360 nm wavelengths were selected in order to analyze the following phenolic compounds: gallic acid, catechin, epicatechin and resveratrol. Phenolic compound peaks were identified via a comparison with the retention time of pure standard. Their concentrations in the samples were calculated via a linear regression curve (y = mx + m) obtained with standard. The standard stock solutions of gallic acid, catechin, epicatechin and resveratrol at 2 mg/mL were prepared in bidistilled water containing 0.004% EDTA and 0.010% sodium bisulfite. The stock solutions were stored at 4 °C, whereas the work solutions (1–20 ng/mL) were obtained daily and progressively by diluting the stock solutions in mobile phase A. The standards of gallic acid, catechin, epicatechin and resveratrol were purchased from Sigma−Aldrich (Milan, Italy).

### 3.6. Antimicrobial Susceptibility Testing

The culture media and chemicals were purchased from Sigma−Aldrich (Milan, Italy). They were: Mueller−Hinton broth (MHB), Rose Bengal Chloramphenicol Agar (RBCA), Roswell Park Memorial Institute (RPMI) 1640, Sabouraud Dextrose Agar (SDA), 2,3,5-Triphenyl-Tetrazolium Chloride (TTC), Tryptic Soy Agar (TSA), morpholinepropanesulphonic acid (MOPS), Ciprofloxacin, Fluconazole and Griseofulvin.

The in vitro antimicrobial activity of n-hexane (n-hx), ethyl acetate (EtOAc) and methanolic extracts (MeOH) of *T. goniospermum* fruiting bodies and mycelia were assessed against eight bacterial strains (CLSI M07-A9), namely *E. coli* (ATCC 10536), *E. coli* (PeruMycA 2), *E. coli* (PeruMycA 3), *B. cereus* (PeruMycA 4), *P. aeruginosa* (PeruMyc 5), *B. subtilis* (PeruMyc 6), *S. typhy* (PeruMyc 7), *S. aureus* (ATCC 6538); and 14 yeasts and dermatophytes strains, namely *C. albicans* (YEPGA 6183), *C. tropicalis* (YEPGA 6184), *C. albicans* (YEPGA 6379), *C. parapsilopsis* (YEPGA 6551), *A. crocatum* (CCF 5300), *A. curreyi* (CCF 5207), *A. gypseum* (CCF 6261), *A. insingulare* (CCF 5417), *A. quadrifidum* (CCF 5792), *T. mentagrophytes* (CCF 4823), *T. mentagrophytes* (CCF 5930), *T. rubrum* (CCF 4933), *T. rubrum* (CCF 4879) and *T. tonsurans* (CCF 4834).

Two yeast strains, *Candida parapsilosis* (ATCC 22019) and *C. krusei* (ATCC 6258), were used as quality controls (CLSI 2008a, b; CLSI 2012; CLSI 2017) for the antifungal assays.

Voucher microbial cultures were maintained in the PeruMyc culture collection of the Department of Chemistry, Biology and Biotechnology (DCBB) (University of Perugia, Italy) and are available upon request.

The determination of Minimum Inhibitory Concentration (MIC) was performed according to the methods described in our earlier studies [31]. Ciprofloxacin was used as the standard for antibacterial activity. Fluconazole and griseofulvin were used as the controls for antifungal agents.

### 3.7. Bioinformatics Analysis

Chemical structures were prepared and converted in .mol files using ChemSketch software. A compound-target analysis was also conducted through the bioinformatics platform STITCH (http://stitch.embl.de/cgi/network.pl), with the aim to predict microbial targets. Regarding the docking analysis, the routine steps for the docking calculations involved the preparation of the inhibitors and the protein. The crystal structures of the proteins were downloaded from the Protein Data Bank (PDB: https://www.rcsb.org/). The PDB codes were: 5I3B (tyrosinase from *Bacillus megaterium*), 5M8P (Human Tyrosinase Related Protein 1), 3MKT [multidrug efflux system transporters of *E. coli* (mdtK)], 5Y50 [MATE transporter AtDTX14], 3ESH [*S. aureus* β-lactamase (ORF259)]. In order to prepare the protein for docking calculations, all water molecules and co-crystalized compounds were removed. This step was followed by adding polar hydrogen atoms and neutralization using Autodock4 program (Molinspiration Database). The starting structures of secondary metabolites were optimized to their ground state structures using the AM1 semiempirical method, and the 3D structures were saved in mol2 format. The protein was immersed in a 3D grid box with 60 × 60 × 60 dimensions with 0.375 Å distance between points. The Lamarckian genetic algorithm was used to calculate the docking free energy of 250 conformations for each inhibitor. The docking results were clustered and organized according to the docking free energy. The binding site was localized, and the non-bonding interactions were elucidated using the Discovery Studio 5.0 visualizer.

## 4. Conclusions

In conclusion, all mushroom extracts tested in this study had potent antimicrobial activities. Particularly, among the tested extracts, the ethyl acetate extract showed the highest efficacy in all proposed experimental paradigms, which could be related, albeit partially, to the content of catechin. The bioinformatics analyses also suggested interactions between this compound and specific microbial proteins involved in the resistance to chemotherapeutic drugs, thus suggesting innovative pharmacological applications of *T. goniospermum* extracts.

## Figures and Tables

**Table 1:** Phenolic compounds in n-hx, EtOAc, MeOH extracts of *Tricholosporum goniospermum* fruiting bodies and mycelia.

*T. goniospermum* Parts	Solvents	Gallic Acid	Catechin	Epicatechin	Resveratrol
		**µg/mL**	**µg/mL**	**µg/mL**	**µg/mL**
	n-hex	0.96 ± 0.5	0.00	0.00	0.00
Fruiting bodies	EA	0.09 ± 0.01	0.03 ± 0.01	0.00	0.00
	MeOH	0.65 ± 0.03	0.00	0.00	0.00
	n-hex	0.95 ± 0.3	0.00	0.00	0.00
Mycelia	EA	2.18 ± 0.11	0.00	0.00	0.00
	MeOH	3.95 ± 0.13	0.00	0.00	0.00

**Table 2:** Total phenolic and antioxidant properties of the tested extracts.

*T. goniospermum* Parts	Solvents	TPC	DPPH	CUPRAC	FRAP
		**(mg GAE/g)**	**(mg TE/g)**	**(mg TE/g)**	**(mg TE/g)**
	n-hex	10.91 ± 0.56	9.35 ± 0.16	53.92 ± 1.31	14.86 ± 1.21
Fruiting bodies	EA	70.51 ± 0.06	88.82 ± 1.47	307.71 ± 3.83	134.06 ± 1.50
	MeOH	14.87 ± 0.79	17.69 ± 0.95	131.52 ± 0.67	20.54 ± 0.77
	n-hex	9.63 ± 0.22	7.53 ± 0.70	54.35 ± 0.92	15.15 ± 1.62
Mycelia	EA	33.74 ± 0.80	29.93 ± 3.54	155.31 ± 1.85	74.26 ± 1.79
	MeOH	7.39 ± 0.14	7.74 ± 0.69	129.60 ± 0.56	12.94 ± 1.33

GAE: Gallic acid equivalent; TE: Trolox equivalent; Values are reported as mean ± S.D.

**Table 3:** Enzyme inhibitory effects of the tested extracts.

*T. goniospermum* Parts	Solvents	AChE Inhibition	BChE Inhibition	Tyrosinase Inhibition	Amylase Inhibition
		(mg GALAE/g)	(mg GALAE/g)	(mg KAE)	(mmol ACAE/g)
	n-hex	NA	5.48 ± 0.03	83.80 ± 1.45	0.55 ± 0.01
Fruiting bodies	EA	NA	26.78 ± 0.21	554.30 ± 9.41	2.97 ± 0.10
	MeOH	NA	5.07 ± 0.02	48.48 ± 0.07	0.21 ± 0.01
	n-hex	NA	9.14 ± 0.07	127.76 ± 0.73	0.73 ± 0.02
Mycelia	EA	NA	25.21 ± 0.24	412.81 ± 1.39	2.25 ± 0.07
	MeOH	NA	2.61 ± 0.01	28.17 ± 0.39	0.17 ± 0.01

GALAE: Galatamine equivalent; KAE: Kojic acid equivalent; ACAE: Acarbose equivalent; NA: not active. Values are reported as mean ± S.D.

**Table 4:** Minimal inhibitory concentrations (MICs) of *Tricholosporum goniospermum* n-hexane, ethyl acetate and methanol extracts, and ciprofloxacin towards selected Gram-negative and Gram-negative bacteria.

Bacterial Strain (ID)	Extract Typology	Minimum Inhibitory Concentration (MIC)			
		n-hex (mg mL^−1^) *	EA (mg mL^−1^) *	MeOH (mg mL^−1^) *	Ciprofloxacin (µg mL^−1^) **
Gram−					
*E. coli* (ATCC 10536)	mycelia	0.157 (0.125–0.25)	0.099 (0.0625–0.125)	0.099 (0.0625–0.125)	<0.12
	fruiting bodies	0.198 (0.125–0.25)	0.157 (0.125–0.25)	0.198 (0.125–0.250)	
*E. coli* (PeruMycA 2)	mycelia	0.315 (0.25–0.5)	0.157 (0.125–025)	0.198 (0.125–0.25)	1.23 (1.95–0.98)
	fruiting bodies	0.396 (0.25–0.5)	0.315 (0.25–0.5)	0.315 (0.25–0.5)	
*E. coli* (PeruMycA 3)	mycelia	0.396 (0.25–0.5)	0.157 (0.125–0.250)	0.198 (0.125–0.250)	0.62 (0.98–0.49)
	fruiting bodies	0.315 (0.125–0.25)	0.198 (0.125–0.250)	0.315 (0.25–0.5)	
*P. aeruginosa* (PeruMyc 5)	mycelia	0.63 (0.5–1)	0.315 (0.25–0.5)	0.396 (0.25–0.5)	1.23 (1.95–0.98)
	fruiting bodies	0.79 (0.05–1)	0.396 (0.25–0.5)	0.63 (0.5–1)	
*S. typhy* (PeruMyc 7)	mycelia	0.79 (0.5–1)	0.315 (0.25–0.5)	0.62 (0.5–1)	0.38 (0.49–0.24)
	fruiting bodies	>1	0.62 (0.5–1)	0.79 (0.5–1)	
Gram+					
*B. cereus* (PeruMycA 4)	mycelia	0.198 (0.125–0.5)	0.099 (0.0625–0.125)	0.157 (0.125–0.250)	<0.12
	fruiting bodies	0.314 (0.25–0.5)	0.157 (0.125–0.250)	0.099 (0.0625–0.125)	
*B. subtilis* (PeruMyc 6)	mycelia	0.315 (0.125–0.25)	0.078 (0.062–0.125)	0.198 (0.125–0.25)	<0.12
	fruiting bodies	0.396 (0.25–0.5)	0.157 (0.125–0.25)	0.314 (0.25–0.5)	
*S. aureus* (ATCC 6538)	mycelia	0.396 (0.25–0.5)	0.157 (0.125–0.25)	0.198 (0.125–0.25)	0.62 (0.98–0.49)
	fruiting bodies	0.63 (0.5–1)	0.198 (0.125–0.25)	0.315 (0.25–0.5)	

* MIC values are reported as the geometric means of three independent replicates (*n* = 3); MIC ranges are reported within brackets. ** MIC values and ranges for fluconazole are expressed as μg mL^−1^. In case no growth was observed at the lowest concentration tested, MIC values are reported as < [lowest concentration tested].

**Table 5:** Minimal inhibitory concentrations (MICs) of *T. goniospermum* n-hexane, ethyl acetate and methanol extracts, and fluconazole towards selected yeasts.

Yeasts (ID)	Extract Typology	Minimum Inhibitory Concentration (MIC)			
		n-hex (mg mL^−1^) *	EA (mg mL^−1^) *	MeOH (mg mL^−1^) *	Fluconazole (µg mL^−1^) **
*C. albicans* (YEPGA 6183)	mycelia	0.314 (0.25–0.5)	0.099 (0.0625–0.150)	0.157 (0.125–0.250)	2
	fruiting bodies	0.396 (0.25–0.5)	0.198 (0.125–0.250)	0.314 (0.25–0.5)	
*C. tropicalis* (YEPGA 6184)	mycelia	0.157 (0.125–0.25)	0.099 (0.0625–0.125)	0.099 (0.0625–0.125)	2
	fruiting bodies	0.198 (0.125–0.25)	0.157 (0.125–0.250)	0.198 (0.125–0.250)	
*C. albicans* (YEPGA 6379)	mycelia	0.099 (0.0625–0.125)	0.051 (0.031–0.0625)	0.099 (0.0625–0.125)	1
	fruiting bodies	0.157 (0.125–0.25)	0.198 (0.125–0.250)	0.198 (0.125–0.250)	
*C. parapsilopsis* (YEPGA 6551)	mycelia	0.314 (0.25–0.5)	0.079 (0.125–0.0625)	0.157 (0.125–0.25)	4
	fruiting bodies	0.396 (0.25–0.5)	0.099 (0.0625–0.125)	0.198 (0.125–0.250)	

* MIC values are reported as the geometric means of three independent replicates (*n* = 3); MIC ranges are reported within brackets. ** MIC values and ranges for fluconazole are expressed as μg mL^−1^. In case no growth was observed at the lowest concentration tested, MIC values are reported as < [lowest concentration tested].

**Table 6:** Minimal inhibitory concentrations (MICs) of *T. goniospermum* n-hexane, ethyl acetate and methanol extracts, and griseofulvin towards selected dermatophytes.

Dermatophytes (ID Strain)	Extract Typology	Minimum Inhibitory Concentration (MIC)			
		n-hex (mg mL^−1^) *	EA (mg mL^−1^) *	MeOH (mg mL^−1^) *	Griseofulvin (µg mL^−1^) **
*A. crocatum* (CCF 5300)	mycelia	0.25 (0.125–0.25)	0.084 (0.031–0.062)	0.157 (0.125–0.25)	>8
	fruiting bodies	0.314 (0.25–0.5)	0.46 (0.62–0.125)	0.198 (0.125–0.25)	
*A. curreyi* (CCF 5207)	mycelia	0.794 (0.5–1)	0.157 (0.125–0.25)	0.363 (0.62–0.125)	>8
	fruiting bodies	>1	0.315 (0.25–0.5)	0.39 (0.031–0.62)	
*A. gypseum* (CCF 6261)	mycelia	0.397 (0.25–0.5)	0.099 (0.62–0.125)	0.049 (0.031–0.062)	1.587 (1–2)
	fruiting bodies	0.397 (0.25–0.5)	0.181 (0.031–0.062)	0.25 (0.125–0.5)	
*A. insingulare* (CCF 5417)	mycelia	0.794 (0.5–1)	0.194 (0.125–0.5)	0.794 (0.5–1)	>8
	fruiting bodies	>1	0.623 (0.5–1)	>1	
*A. quadrifidum* (CCF 5792)	mycelia	0.623 (0.5–1)	0.198 (0.125–0.25)	0.315 (0.25–0.5)	>8
	fruiting bodies	>1	0.623 (0.5–1)	0.794 (0.5–1)	
*T. mentagrophytes* (CCF 4823)	mycelia	0.315 (0.25–0.5)	0.157 (0.125–0.25)	0.315 (0.25–0.5)	2.52 (2–4)
	fruiting bodies	0.623 (0.5–1)	0.194 (0.125–0.25)	0.397 (0.5–1)	
*T. mentagrophytes* (CCF 5930)	mycelia	0.794 (0.5–1)	0.57 (0.125–0.25)	0.63 (0.5–1)	3.174 (2–4)
	fruiting bodies	>1	0.198 (0.125–0.25)	>1	
*T. rubrum* (CCF 4933)	mycelia	0.397 (0.25–0.5)	0.049 (0.031–062)	0.039 (0.031–0.062)	1.26 (1–2)
	fruiting bodies	0.794 (0.5–1)	0.198 (0.125–0.25)	0.315 (0.25–0.5)	
*T. rubrum* (CCF 4879)	mycelia	0.397 (0.25–0.5)	0.049 (0.031–0.062)	0.315 (0.25–0.5)	3.175 (2–4)
	fruiting bodies	0.794 (0.5–1)	0.157 (0.125–0.25)	0.623 (0.5–1)	
*T. tonsurans* (CCF 4834)	mycelia	0.397 (0.25–0.5)	0.039 (0.031–0.062)	0.099 (0.062–0.125)	0.198 (0.125–0.25)
	fruiting bodies	0.623 (0.5–1)	0.314 (0.25–0.5)	0.397 (0.25–0.5)	

* MIC values are reported as the geometric means of three independent replicates (*n* = 3); MIC ranges are reported within brackets. ** MIC values and ranges for griseofulvin are expressed as μg mL^−1^. In case no growth was observed at the lowest concentration tested, MIC values are reported as < [lowest concentration tested].
